# IFN-λ Modulates the Migratory Capacity of Canine Mammary Tumor Cells via Regulation of the Expression of Matrix Metalloproteinases and Their Inhibitors

**DOI:** 10.3390/cells10050999

**Published:** 2021-04-23

**Authors:** Rafał Pingwara, Daria Kosmala, Natalia Woźniak, Arkadiusz Orzechowski, Joanna Mucha

**Affiliations:** Department of Physiological Sciences, Institute of Veterinary Medicine, Warsaw University of Life Sciences-SGGW, 02-776 Warsaw, Poland; rafal_pingwara@sggw.edu.pl (R.P.); kosmala.daria92@gmail.com (D.K.); n.wozniak95@gmail.com (N.W.); orzechowski_arkadiusz@wp.pl (A.O.)

**Keywords:** IL-28, IL-29, breast cancer, STAT, interferon-stimulated-genes, Akt, ERK, apoptosis, proliferation, metastasis

## Abstract

Interactions between neoplastic and immune cells taking place in tumors drive cancer regulatory mechanisms both in humans and animals. IFN-λ, a potent antiviral factor, is also secreted in the tumor; however, its role in tumor development is still unclear. In our study, we investigate the influence of IFN-λ on the canine mammary tumor (CMT) cell survival and their metastatic potential in vitro. First, we examined, by Western blot, the expression of the IFN-λ receptor complex in three CMT cell lines (P114, CMT-U27 and CMT-U309). We showed that only two cell lines (P114 and CMT-U27) express both (IL-28RA and IL-10Rb) receptor subunits and respond to IFN-λ treatment by STAT phosphorylation and the expression of interferon-stimulated genes. Using MTT, crystal violet and annexin-V assays, we showed a minimal role of IFN-λ in CMT viability. However, IFN-λ administration had a contradictory effect on cell migration in the scratch test, namely, it increased P114 and decreased CMT-U27 motility. Moreover, we demonstrated that this process is related to the expression of extracellular matrix metalloproteinases and their inhibitors; furthermore, it is independent of Akt and ERK signaling pathways. To conclude, we showed that IFN-λ activity is reliant on the expression of two receptor subunits and tumor type, but further investigations are needed.

## 1. Introduction

The mammary tumor is the second most frequently diagnosed tumor in dogs and the most common in non-spayed females. Canine mammary tumors (CMT) develop spontaneously with age, are sex hormone-dependent, and might be the consequence of *TP53* and *BRCA1* gene mutations [1,2]. The same etiological factors have been identified in human breast cancer. Moreover, treatment approaches for canine and human mammary gland tumors are very similar, including surgical resection, chemotherapy, and radiotherapy (or both). For these reasons, the CMT is considered to be a valuable and reliable human breast cancer model [3]. In addition, other companion animals, for example, cats, are increasingly used as breast cancer models [4]. Many similarities have been demonstrated in the clinical behavior of the disease, etiology, and molecular subtypes of tumors between feline and human breast cancer [5]. Moreover, strong changes in the immune response are observed in dogs and cats, similar to those in humans [4,6,7,8].

The latest research has shown stroma reprogramming during CMT development, similar to human breast cancer. In addition to transformed cells, the CMT environment consists of endothelial cells, fibroblasts, macrophages, lymphocytes and myeloid-derived suppressor cells (MDSC). The cross-talk between cancer cells and normal cells may occur via direct cell–cell contact or by the secretion of various molecules, for example, growth factors, cytokines and chemokines, to the extracellular matrix [9,10,11,12]. In our previous study, we showed an increased presence of MDSC in the tumors and blood of CMT patients. We also observed that these cells can secrete IFN-λ [13].

Interferons are among the most important mediators of anti-viral immune response, and currently, three types of these proteins have been identified. Type I IFNs are the largest group of IFNs, which include 17 different genes encoding these proteins. On the contrary, the II type of interferons is represented by only one protein, IFN-γ [14]. In 2003, a new type of IFNs was identified and classified as INF-λ [15,16]. Thus far, four genes encoding these proteins have been identified in humans [17]. They have also been identified in other species inter alia mice, chickens, cattle and bats [18,19,20,21]. Yet, two genes encoding INF-λ have been identified in dogs [22]. The recombinant CaIFN-λ1 and CaIFN-λ3 proteins have been synthesized, and their antiviral properties have been demonstrated against vesicular stomatitis virus, canine parvovirus and influenza virus [22,23,24].

Aside from its antiviral properties, IFN-λ was reported to be produced in the tumor by various types of cells, mainly plasmacytoid dendritic cells [25,26]. It was also documented that IFN-λ is produced by cancer cells themselves, for example, by the human colon, liver and breast cancer [27,28,29]. However, data regarding this cytokine’s role in tumorigenesis are limited, and contradictory properties are described [30]. There are several reports showing that IFN-λ induces apoptosis and inhibits cancer cell proliferation. Moreover, IFN-λ intensifies innate and adaptive anti-tumor immune response [31]. On the other hand, few studies have demonstrated the pro-tumor activity of this cytokine. IFN-λ enhances multiple myeloma cell proliferation and increases the metastatic potential of mammary and bladder cancer [13,32,33,34,35]. However, the molecular basis of its action and role in different types of tumor are unclear, and more research is needed.

Therefore, the goal of our study was to broaden the knowledge on the role of the IFN-λ in canine mammary tumor progression. We carried out a series of in vitro experiments to evaluate the molecular mode of IFN-λ action on the CMT cells. We examined the viability, proliferation and programmed cell death of CMT cells treated with IFN-λ. Moreover, the impact of the IFN-λ on the migratory capacity of CMT cells was examined, and the probable mechanism of cell motility regulation was proposed.

## 2. Materials and Methods

### 2.1. Canine Mammary Tumor Cell Lines and Culture Conditions

We carried out our research on three canine mammary tumor cell lines. The anaplastic cancer cell line, P114, was kindly donated by Dr. Gerard Rutteman (Utrecht University, Utrecht, The Netherlands). A simple carcinoma cell line (CMT-U27) and a spindle cell mammary tumor cell line (CMT-U309) were established by Prof. Dr. Eva Hellmén (Swedish University of Agricultural Sciences, Uppsala, Sweden).

All cell lines were cultured in RPMI 1640 medium supplemented with 10% of heat-inactivated fetal bovine serum, penicillin-streptomycin (50 IU/mL) under standard conditions for mammalian cells: 37 °C, 5% CO_2_, and 95% humidity. All reagents were purchased from ThermoFisher Scientific, Waltham, MA, USA. The medium was replaced every 2–3 days, and cells were passaged via trypsinization. All cell cultures were routinely checked for mycoplasma contamination by vital staining with Hoechst (Sigma Aldrich St., St. Louis, MO, USA).

To limit the influence of other serum components or the synergistic effect with IFN-λ on investigation results, before each experiment, the growth medium (10% FBS) was replaced with a control medium (supplemented with 2% BSA).

### 2.2. Cell Viability and Proliferation Assay

To assess the viability (MTT assay) and proliferation (crystal violet—CV staining), cells were seeded on a 96-well plate at a density of 2 × 10^4^ cells per well. After 24 h of culture, growth medium was replaced by the control medium (RMPI + 2%BSA) and cultured for the next 24 h. Subsequently, for another 24 h, the cells were treated with human recombinant IFN-λ1 or IFN-λ2 (R&D Systems, Minneapolis, MN, USA) diluted in the control medium at the following concentrations: 1, 10, 100, 500 and 1000 ng/mL. Cells cultured with the control medium without cytokines were used as the control. After incubation, the medium was removed, and cell viability in the MTT assay and cell proliferation by crystal violet (CV) staining were examined.

The MTT (3-(4,5-dimethylthiazol-2-yl)-2,5-diphenyltetrazolium bromide) salt (Sigma-Aldrich, St. Louis, MO, USA) was prepared in RPMI phenol red-free medium, and 100 µL was added to each well at a concentration of 1mg/mL and incubated for 2 h. Cells with formed formazan crystals were lysed in 100 µL DMSO. The absorbance of dissolved formazan was measured at 570 nm using a multiplate reader type Infinite 200 (Tecan, Mannedorf, Switzerland).

For CV staining, cells were washed with PBS and fixed twice with 75% and 100% (*v*/*v*) ice-cold methanol for 20 min at 4 °C. Then, cells were stained with 0,2% (*w*/*v*) CV/H2O for 20 min at room temperature. Subsequently, they were gently washed with distilled H2O, air-dried, and lysed with 100 µL DMSO. The absorbance for CV was measured at 570 nm with multiplate reader type Infinite 200 (Tecan, Mannedorf, Switzerland).

### 2.3. Apoptosis Assay

For cell death examination, Annexin-V and DRAQ7 staining and flow cytometry analyses were applied. The CMT cells were cultured on a 48-well plate; 24 h prior to the experiment, the growth medium was replaced with the control medium. Cells were treated with IFN-λ1 and IFN-λ2 at the concentration of 100 ng/mL for 72 h. For analysis, cells were harvested by trypsinization, centrifuged with supernatant and stained using Annexin V (Invitrogen, ThermoFisher Scientific, Waltham, MA, USA) according to the manufacturer’s protocol. Finally, DRQ7 was added to each tube (1:1000 dilution), and samples were analyzed by flow cytometry (BD FACS Aria II, Becton Dickinson, Franklin Lakes, NJ, USA). Data were analyzed with FlowJo software (TreeStar Inc., Ashland, OR, USA). Cells were gated for their size and granularity, and doublets were excluded from the analysis. Cells in early apoptosis with exposed phosphatidylserine bound to Annexin V-FITC only. Late apoptotic cells were labeled with both Annexin V-FITC and DRAQ7, while dead cells other than apoptotic were stained with DRAQ7 only.

### 2.4. Real-Time Quantitative PCR

To examine the mRNA expression of interferon-stimulated genes (ISG) and extracellular matrix metalloproteinases (MMPs) and their tissue inhibitors (tissue inhibitors of metalloproteinases—TIMPs) in CMT cells treated with IFN-λ1 or IFN-λ2, real-time quantitative PCR reaction was carried out. The CMT cells were cultured on a 12-well plate, and 24 h before the experiment, growth medium was replaced with the control medium. Subsequently, cells were treated with IFN-λ1 or IFN-λ2 for 12 h (ISG expression) and 24 h (MMPs and TIMPs). After incubation, RNA was isolated with the use of the Total RNA kit (A&A Biotechnology, Gdynia, Poland) according to the manufacturer’s recommendations and stored at −80 °C. The concentration of isolated RNA was determined using NanoDrop 2000 (NanoDrop Technologies, Waltham, MA, USA). To eliminate DNA, the samples with adequate quantity of RNA were treated with DNaseI. According to the manufacturer’s protocol, High-Capacity cDNA Reverse Transcription Kits (ThermoFisher Scientific, Waltham, MA, USA) were used for cDNA synthesis in the Mastercycler thermal cycler (Eppendorf, Hamburg, Germany). For examination of *ISG15* and *MX1* expression, the primers reported by Fan et al. 2014 were used [24]. For the *OAS-1* expression measurement, due to the fact that there was no product synthesis observed with the primer for OAS reported by Fan et al. 2014, we designed the primers de novo using PRIMER3 software (free online access, http://bioinfo.ut.ee/primer3-0.4.0/, accessed on 23 July 2019). Primers used in the study for *MMP-2*, *MMP-9*, *MMP-13*, *TIMP-1*, *TIMP-2* and *TIMP-3* were reported in [36]. All primers were checked using an Oligo Calculator (free online access, http://biotools.nubic.northwestern.edu/OligoCalc.html, accessed on 23 July 2019) and Primer-Blast (NCBI database, https://www.ncbi.nlm.nih.gov/tools/primer-blast/, accessed on 23 July 2019). The sequences of primers used for qPCR are listed in Table 1.

The qPCR reaction was performed with the use of SYBR Green and the Stratagene Mx3005P QPCR System (Agilent Technologies, Santa Clara, CA, USA) in sequence: an initial denaturation step at 50 (2 min) and 95 °C (2 min), followed by 35 cycles at 95 °C (15 s), an annealing step at 58 °C (15 s), and 72 °C extensions (1 min). RPS19 gene was used as housekeeping for normalization of the examined gene expression. The comparative Ct method as 2^−ΔΔCt^, (ΔCt  =  Ctreference − Cttarget) was applied to calculate the relative mRNA expression. The results were presented as normalized to control cells.

### 2.5. Western Blot

To measure the phosphorylation levels of signal transducer and activators of transcription proteins (STAT1 and STAT3), CMT cell lines were stimulated with IFN-λ1 or IFN-λ2 (100 ng/mL) for 30 min. Protein extraction from CMT cells was performed using NE-PER™ Nuclear and Cytoplasmic Extraction Reagents (ThermoFisher Scientific, Waltham, MA, USA) according to the manufacturer’s protocol additionally supplemented with Protease and Phosphatase inhibitors (ThermoFisher Scientific, Waltham, MA, USA).

To determine the expression level of the IFN-λ receptor and Akt and ERK kinases, total protein was isolated from CMT cell lines using RIPA buffer (ThermoFisher Scientific, Waltham, MA, USA) supplemented with Protease and Phosphatase inhibitors. For examination of Akt and ERK activation in CMT cells, the cells prior to protein isolation were treated with IFN-λ1 or IFN-λ2 for 5, 10, 15, 20 and 30 min.

Protein concentration was determined by BCA protein assay (ThermoFisher Scientific, Waltham, MA, USA). Proteins (nuclear fraction—10 µg; total fraction—30 µg) were resolved using SDS-PAGE and transferred onto nitrocellulose membranes (ThermoFisher Scientific, Waltham, MA, USA). Membranes were blocked with 5% BSA in TBS buffer containing 0.1% Tween 20 for 90 min. Subsequently, membranes were incubated overnight with the primary anti-canine antibodies (or antibodies showing cross-reactivity with canine) at 4°C (antibodies and used concentrations are listed in Table 2).

Next, membranes were washed three times with TBS containing 0.1% Tween 20 and incubated for 1 h with secondary antibodies conjugated with the appropriate infrared (IR) fluorophore IRDye 800 CW or IRDye 680 RD (Li-Cor: cat. no.: 926-68072, 926-68074, 926-32213) at a dilution of 1:5000 at room temperature. To analyze protein expression, the Hemidoc Imaging System (Bio-Rad Laboratories Inc., Hercules, CA, USA) was used. Quantification of the integrated optical density (IOD) was obtained using the ImageJ software (National Institutes of Health, Bethesda, MD, USA). The results were presented as normalized to control cells.

### 2.6. Scratch Test (Wound Healing Assay)

The cells were seeded on a 24-well plate and cultured for 24 h in a growth medium containing different concentrations of IFN-λ1 or IFN-λ2 (1, 10 and 100 ng/mL) to reach a high-density monolayer of cells. Then, for the next 24 h, cells were treated with IFN-λ1 or IFN-λ2 in the same concentrations (1, 10 and 100 ng/mL) in the control medium. Subsequently, the monolayer was wounded by scratching the surface as uniformly and straight as possible with a pipette tip (100 µL), then medium with detached cells was removed, and cells were washed with PBS and cultured in control medium for 24 h. The images of cells invading the scratch were captured at indicated time points (0, 3, 6, 12 and 24 h) using a phase-contrast microscope (IX 70 Olympus Optical Co., Hamburg, Germany).

The pictures were analyzed using a computer-assisted image analyzer ImageJ 1.50b (National Institutes of Health, Bethesda, MD, USA). Each picture was analyzed independently by two people. The migration potential of the neoplastic cells of the CMT cell lines was expressed as a percentage of atresia of the wound concerning its area at time “0”, which was considered completely not grown (0%).

### 2.7. Statistical Analysis

All experiments were performed in triplicate. Prism version 5.00 software (GraphPad Software, San Diego, CA, USA) was applied to obtain the statistical analysis, and the one-way ANOVA and Dunnett’s HSD post hoc test were used. A *p*-value < 0.05 was regarded as significant while a *p*-value < 0.01 and *p*-value < 0.001 as highly significant.

## 3. Results

### 3.1. Canine Mammary Tumor Cells Express the Receptor for IFN-λ and Respond to This Cytokine

**Figure 1:**
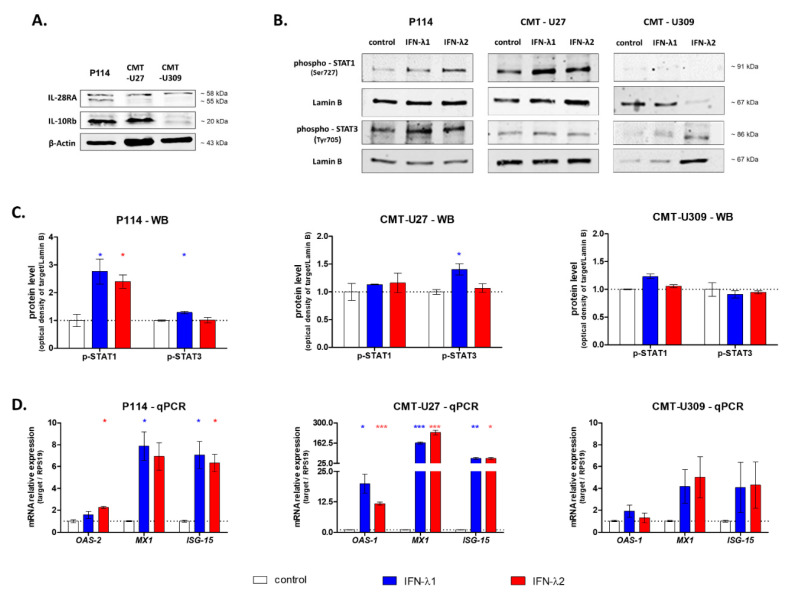
Representative images of Western blot showing the expression of IL-28RA, IL-10Rb and β-actin (loading control) in whole CMT cell lysates (**A**) and phospho-STAT1, phospho-STAT3 and Lamin B (loading control) in the nuclear fraction of CMT cells after 30 min treatment with IFN-λ1 and IFN-λ2 (**B**). The graphs showing the relative phosphorylation of STAT1 and STAT3 in CMT cells after 30 min treatment with IFN-λ1 and IFN-λ2 (**C**). The graphs showing the relative mRNA of ISG expression in CMT cells treated with IFN-λ1 and IFN-λ2 for 12 h (**D**). The results are presented as the mean ± SEM. A one-way ANOVA and Dunnet’s HSD post hoc test were applied. Significance levels are indicated as follows: * *p* < 0.05; ** *p* < 0.01; *** *p* < 0.001.

First, to assess the IFN-λ receptor presence in all three cell lines used in this study, we performed Western blot analysis. We showed the expression of the IL-28RA subunit in all three cell lines; however, CMT-U27 cells do not express the second subunit of the receptor—IL-10Rb (Figure 1A).

To further evaluate the IFN-λ biological activity in CMT cells, two major pathways activated by this cytokine were measured, namely, STAT phosphorylation and interferon-stimulated gene expression. Western blot analysis showed that in P114 cells treated with IFN-λ1, the phosphorylation of STAT1 and STAT3 increased, whereas supplementation with IFN-λ2 enhanced only STAT3 phosphorylation. In the case of CMT-U27 cells, only under IFN-λ1 treatment higher levels of phosphorylation of STAT3 were observed. CMT-U309 cells exhibit the lowest basal phosphorylation of both STATs, and no effect of IFN-λ1 nor IFN-λ2 was detected (Figure 1B,C).

In the next step, we assessed the impact of IFNs-λ on the expression of interferon-stimulated genes. The highest changes in ISG expression were observed in CMT-U27 cells. Almost 200- and 25-fold increases in *MX1* and *ISG-15* expression under both IFN-λ1 and IFN-λ2 treatment, respectively, were observed. In CMT-U27 cells, treatment with both cytokines upregulated the *OAS-2* expression more than 10-fold in comparison to non-treated cells. In P114 cells, both cytokines did not affect the *OAS-2* and *MX1* expression; however, IFN-λ2 upregulated the *OAS-2* expression by 2-fold. For all the remaining genes, more than a 5-fold increase in expression was observed. In CMT-U309, no significant impact of IFN-λ1 and IFN-λ2 treatment on *ISG* expression was observed (Figure 1D).

### 3.2. IFN-λ Does Not Regulate Canine Mammary Tumor Cell Survival

The cell viability of canine mammary cancer cell lines was examined using three different tests: the MTT, CV and Annexin-V assay.

**Figure 2:**
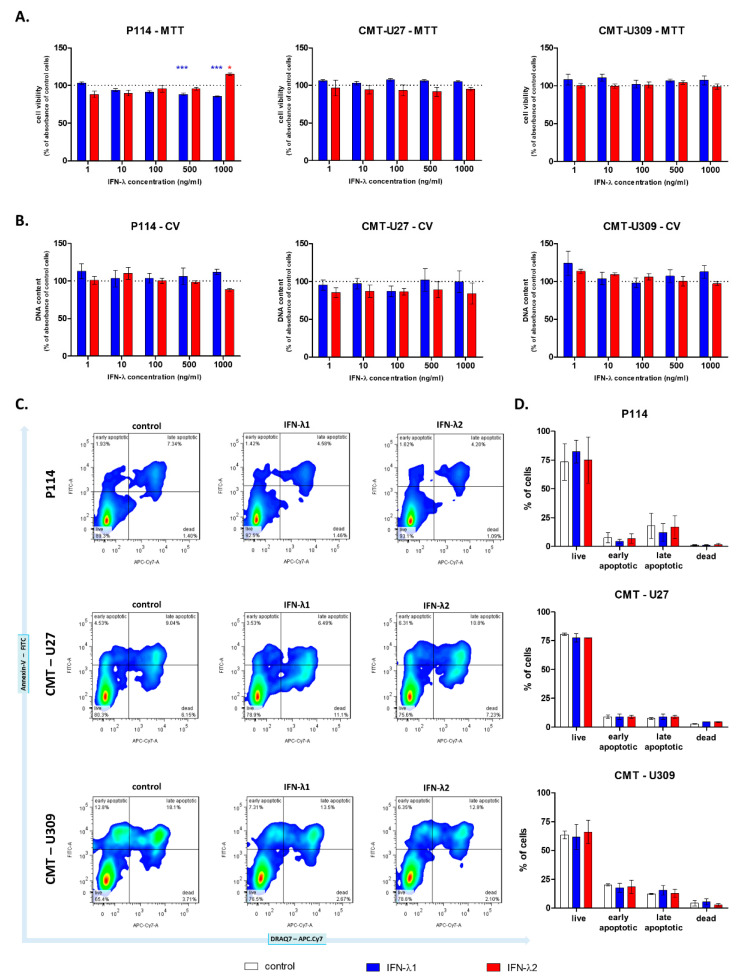
The graphs showing relative CMT cell viability (**A**) and their DNA content (**B**). Representative cytograms (**C**) and graphs (**D**) showing CMT cell mortality. The results are presented as the mean ± SEM. A one-way ANOVA and Dunnet’s HSD post hoc test were applied. Significance levels are indicated as follows: * *p* < 0.05; *** *p* < 0.001.

The MTT assay did not show an effect of IFN-λ1 or IFN-λ2 on CMT cell respiration, except for the P114 cells treated with the highest cytokine concentrations. The metabolic activity of these cells decreased to approximately 90% and 85% after 24 h incubation with IFN-λ1 in 500 and 100 ng/mL, respectively. However, treatment with IFN-λ2 at 1000 ng/mL increased P114 cell respiration by 15% (Figure 2A). The CV assay revealed that IFN-λ does not affect the level of cellular DNA of CMT cells (Figure 2B). Moreover, the assessment of programmed cell death did not show any differences between IFN-λ-treated cells and cells cultured in control medium (Figure 2C,D).

### 3.3. IFN-λ Modulates Canine Mammary Tumor Cell Migration

The scratch test (wound healing assay) was performed to characterize the effect of IFN-λ1 and IFN-λ2 on the migratory abilities of canine cancer cells.

**Figure 3:**
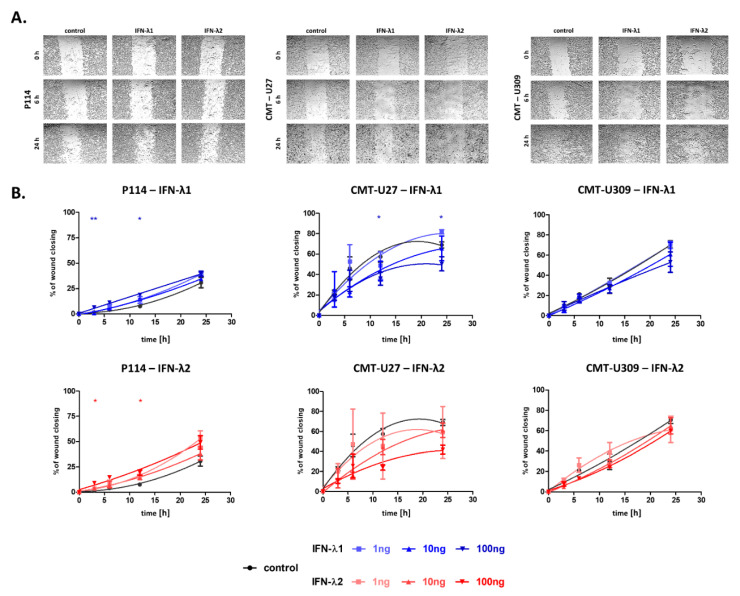
Pictures of the wound-healing processes of CMT cells under IFN-λ1 or IFN-λ2 treatment at the concentration of 100 ng/mL at the selected time points after scratching (**A**) The graphs showing the wound healing speed in time after scratching in cells cultured in control conditions or treated with IFN-λ1 or IFN-λ2 (**B**). The results are presented as the mean ± SEM. A one-way ANOVA and Dunnet’s HSD post hoc test were applied. The statistical differences found between control cells and cells treated with IFN-λ at 100 ng/mL concentration are presented by asterisks. Significance levels are indicated as follows: * *p* < 0.05; ** *p* < 0.01.

In P114 cells, the promoting effect of IFN-λ1 and IFN-λ2 on their motility was observed. At 3 and 12 h after scratching, a decreased “wound” area was observed in P114 cells treated with cytokines in 100 ng/mL concentrations in comparison to non-treated cells. In P114 cells treated with lower concentrations (1 and 10 ng/mL) of both cytokines, no statistical significance was observed (Figure 3A,B).

Interestingly, we observed the reverse effect of the IFN-λ1 and IFN-λ2 on the CMT-U27 cell migratory capacity in comparison to P114 cells (Figure 3A,B). CMT-U27 cells supplemented with IFN-λ1 in 100 ng/mL concentration at 12 and 24 h after scratching exhibited decreased “wound” closing. In the case of CMT-U27 treated with IFN-λ1 in lower concentrations (1 and 10 ng/mL) and IFN-λ2 in all tested concentrations (1, 10 and 100 ng/mL), no statistical significance was observed (Figure 3A,B).

The migratory abilities of CMT-U309 were not affected by treatment with IFN-λ1 nor IFN-λ2 (Figure 3A,B).

### 3.4. IFN-λ Regulates Extracellular Matrix Metalloproteinases and Their Inhibitors Expression in a Akt- and ERK-Independent Manner

**Figure 4:**
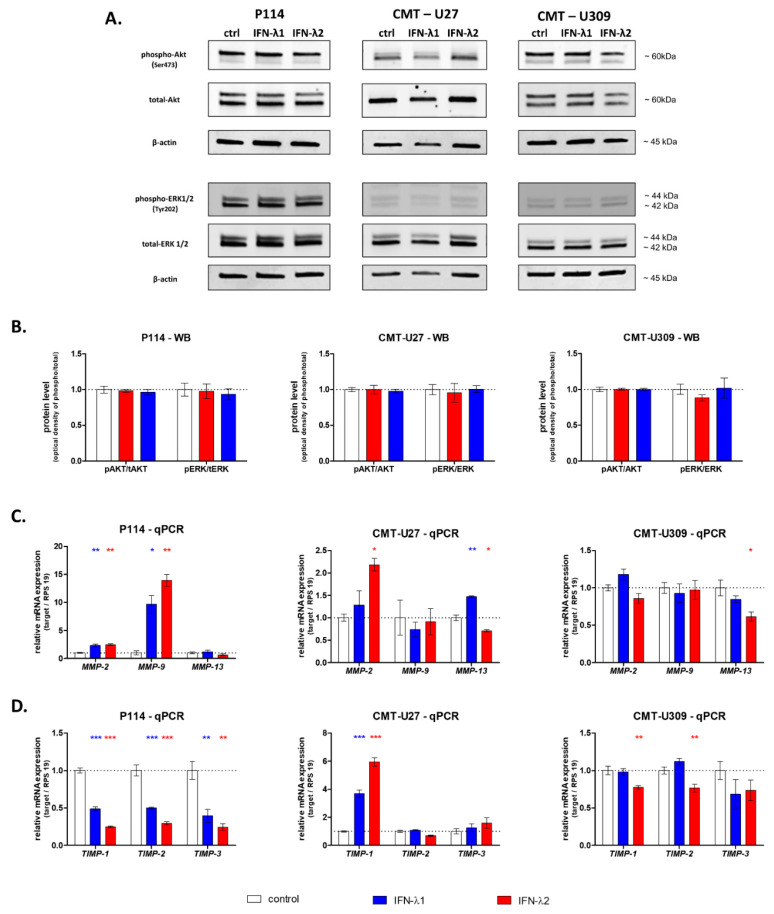
Representative images of Western blot showing the expression of phospho-Akt and Akt, phospho-ERK1/2 and total ERK1/2 and β-actin (loading control) in whole CMT cell lysates (**A**). The graphs showing the relative phosphorylation of Akt and ERK1/2 in CMT cells (**B**). The graphs showing the relative mRNA of extracellular matrix metalloproteinase expression in CMT cells treated with IFN-λ1 and IFN-λ2 for 24 h (**C**). The graphs showing the relative mRNA of extracellular matrix metalloproteinases inhibitor expression in CMT cells treated with IFN- λ1 and IFN-λ2 for 24 h (**D**). The results are presented as the mean ±SEM. A one-way ANOVA and Dunnet’s HSD post hoc test were applied. Significance levels are indicated as follows: * *p* < 0.05; ** *p* < 0.01; *** *p* < 0.001.

To investigate the mechanism of IFN-λ action on CMT cell migration, first, the activation of Akt and ERK signaling pathways was examined by Western blot. Our study showed that CMT treatment with IFN-λ1 or IFN-λ-2 does not influence the phosphorylation of Akt or ERK (Figure 4A,B).

Further, using quantitative real-time PCR, we analyzed the impact of IFN-λ treatment on the expression of extracellular matrix metalloproteinases (MMPs) and their tissue inhibitors (TIMPs). The 24 h stimulation of P114 cells with IFN-λ1 and IFN-λ2 upregulated the expression of *MMP-2* and *MMP-9* (Figure 4C). Simultaneously, a strong downregulation of the expression of their tissue inhibitors *TIMP-1* and *TIMP2* was detected (Figure 4D). Moreover, we observed an increased expression of *TIMP-3* in P114 cells treated with IFN-λ1 and IFN-λ2 (Figure 4D).

The expression of genes encoding metalloproteinases in CMT-U27 cells showed a significant upregulation of *MMP-2* after IFN-λ2 treatment and *MMP-13* after both IFN-λ1 and IFN-λ2 supplementation (Figure 4). No significant impact of IFNs-λ on *MMP-9* and IFN-λ2 on *MMP-2* was observed (Figure 4C). However, both cytokines increased the level of *TIMP-1* expression (Figure 4D). With regard to other *TIMPs*, no effects of IFN-λ1 and IFN-λ2 on their expressions were detected (Figure 4D).

In the case of CMT-U309, the expression of the majority of tested genes was not influenced by treatment with both cytokines; however, lower expressions of *MMP-13*, *TIMP-1* and *TIMP-2* were observed in cells incubated with IFN-λ2.

## 4. Discussion

Immunotherapy has become a new, promising prospect in cancer treatment. Both in humans and dogs, the attempts to use T lymphocyte and NK cell transfer, monoclonal antibodies, immune-checkpoint and immunoregulatory protein inhibitors, as well as cytokines, have been reported [37,38,39]. Thus far, only two cytokines have been approved by the Food and Drug Administration for human cancer treatment: IFN-α and IL-2 [40]. The application of IFN-α inhibits tumor development; however, the side effects of this cytokine limit its uses. IFN-α was reported to have multiple side effects, e.g., high effector cell activation, fever, chills, depression, and anorexia [41]. The discovery of group III interferons—interferon lambda—brought new hope for IFN treatment of cancer. Due to the fact that the expression of the IFN-λ receptor is limited mainly to epithelial cells, whereas IFN-α receptor is expressed in the plasma membrane of the majority of cell types, including T cell and endothelium, IFN-λ treatment is considered more specific and safer [31]. However, detailed research about the role of IFN-λ in cancer development is still lacking, and published data suggest both its pro-and anti-tumoral activity. In this study, we used three different subtypes of CMT cell lines, anaplastic cell carcinoma P114, simple carcinoma CMT-U27 and spindle cell carcinoma CMT-U309, to study the impact of IFN-λ on the survival and metastatic potential of CMT in vitro.

First, we examined the expression of the IFN-λ receptor in three CMT cell lines. Our study showed the expression of IL-28RA in all examined cell lines, but a lack of IL-10Rb expression in the CMT-U309 cell line. The study conducted on human breast cancer demonstrated that the high expression of IL-28RA is the favorable marker for patients, and the same trend was observed in IL-10Rb [25,42,43]. Interestingly, the study conducted by Rajakylä et al. showed that CMT-U309 has the highest migratory capacity in comparison to the CMT-U27 line, in accordance with our observations [44]. It was demonstrated in human cells that only the expression of both receptor subunits enables signal transduction induced by IFN-λ. IFN-λ binds first to the IL-28RA chain; then, conformational changes facilitate the recruitment of the IL-10Rb chain, resulting in the formation of the ligand–receptor complex. As a consequence of ligand binding to the receptor, STAT phosphorylation and ISG synthesis occur [14]. Our study showed the increased phosphorylation of STAT1 and STAT3 proteins and the upregulation of ISG expression in P114 cells upon IFN-λ treatment. Such evident changes in STAT activation were not observed in CMT-U27 cells; however, the ISG upregulation in CMT-U27 cells was the highest in all examined cell lines. There are reports showing that ISG expression may be regulated in the non-canonical STAT-independent pathway, such as the activation of Crk-like protein (CrkL)–Ras-related protein 1 (RAP1) and the phosphatidyl-inositol 3-kinase (PI3K), and the mitogen-activated protein kinase (MAPK) signaling pathways [14]. Our study showed that P114 and CMT-U27 cells have high constitutive STAT autophosphorylation. The same phenomenon was observed in human breast cancer cell lines, in particular, in cells with a high grade of malignancy [45]. Generally, STAT1 and STAT3 are highly expressed and considered to be among the most important transcription factors in human breast tumors [46,47]. Moreover, a similar tendency was observed in CMT; the study conducted by Król et al. 2011 showed the correlation between the expression level of p-STAT3 and the degree of tumor malignancy. p-STAT3 expression was observed not only in cancer cells but also in the myeloid cells [48]. A lack of STAT activation and ISG synthesis in CMT-U309 indicates that IL-10Rb is essential, similarly to human cells, for the response to the IFN-λ of canine cells. Moreover, we suspect that more aggressive mammary tumor cells may be less sensitive to IFN-λ. In our previous study in a murine model of mammary tumor, we showed no impact of IFN-λ on the proangiogenic properties of 4T1 cells (highly malignant, the model for IV grade of human breast cancer malignancy) as opposed to EMT6 cells, which are less aggressive [35].

Many studies have reported that IFN-λ inhibits the growth of various types of tumors, for example, melanoma, colorectal cancer and lung cancer, via the induction of apoptosis, proliferation inhibition and cell cycle arrest [49]. Our study showed no significant role of IFN-λ1 and IFN-λ2 in the regulation of CMT-U27 and CMT-U309 cell survival. Similar observations were obtained using human bladder cancer cells under IFN-λ2 treatment [34]. Herein, in P114 cells, only the high dose of IFN-λ1 (500 and 1000 ng/mL) significantly inhibited their cellular respiration. Moreover, P114 cell treatment with IFN-λ2 at the concentration of 1000 ng/mL increased cell viability in the MTT assay. However, this result is surprising; it is similar to that in the study by Novak et al., who showed that IFN-λ1 promotes multiple myeloma B cell proliferation [32]. In our study, no changes in cell survival were observed in proliferation (CV assay) and apoptosis (Annexin-V staining); therefore, we assume that IFN-λ does not influence the apoptosis and proliferation of CMT cells. The recent data showed that IFN-λ may induce the autophagy process in cancer cells [50,51]. Autophagy is a constitutive degradation mechanism ubiquitous in live cells, and its role in cancer cell survival is dual [52]. On the one hand, especially one type of microautophagy—mitophagy, selective mitochondria degradation—downregulates the expression of succinate dehydrogenases and as consequence may lead to declined cell viability [53]. On the other hand, autophagy provides substrates for cell respiration [54]. In addition, it is demonstrated that other IFN types, IFN-α and IFN-γ, may enhance fatty acid oxidation, glycolysis and oxidative phosphorylation, which in turn may influence cellular respiration without changes in cell proliferation or survival [55,56]. To conclude, we did not observe cytostatic or cytotoxic effects of IFN-λ on CMT even when using very high doses of the cytokine.

The acquisition of the motility by neoplastic cells is a key element for metastasizing. In our study, the opposite effect of IFN-λ on the P114 and CMT-U27 cells motility was observed. The extracellular matrix degradation process is related to cell migration. In the P114 cells, IFN-λ intensified cell migration with increased *MMPs* and downregulated *TIMP* expression. In CMT-U27 cells, IFN-λ treatment reduced cell motility and significantly upregulated *TIMP-1* and declined *MMP-13* expression levels. The detailed role of IFN-λ in tumor cell migration is unclear. Increased migratory properties induced by IFN-λ were reported in human bladder, canine and mouse mammary cancer cells [13,34,35]. Opposite results were obtained in human osteosarcoma cells [51]. Moreover, tumors from patients with an advanced stage of bladder cancer have a higher expression of IFN-λ2 [33]. The increased level of IFN-λ2 expression was also observed in MDSC isolated from canine blood with advanced CMT as well as in MDSC isolated from melanoma metastatic foci in lungs [13,35]. However, the high expression of IFN-λ in human breast cancer is described as a positive marker for patients [25]. Our observations regarding the involvement of MMPs in the modulation of migration are similar to the research reported by Lee et al. In human bladder cells supplemented with IFN-λ2, the authors observed an increased expression and activity of MMP-2 and MMP-9 in an ERK-dependent manner [34]. However, our research showed that in CMT, IFN-λ does not influence ERK signaling pathway activation. Our data comply with results obtained by Zhou et al. in the human Burkitt’s lymphoma B cells. The authors did not observe an effect of IFN-λ on ERK activation in lymphoma cells [57]. Similarly, Guenterberg et al. did not observe the activation of ERK or the Akt signaling pathway in melanoma cells upon IFN-λ2 treatment [58]. We hypothesize that MMP and TIMP expression in CMT cells under IFN-λ treatment may be regulated by STAT activation or another, not yet identified pathway [59,60]. MMPs are commonly expressed in CMTs, and similarly to STAT, these enzymes correlate positively with the grade of tumor malignancy [36].

We speculate that the pro or anti-tumor activity of IFN-λ may depend on the origin of the tumor, its histological type and the stage of development. Depending on the type of tumor-infiltrating immune cells and established cytokine network, the immune system may have an inhibitory or promoting effect on cancer growth. An extensive study of its dual role in tumor development and anti-tumor immune response needs to be verified in in vivo studies.

## 5. Conclusions

To summarize, the expression of two IFN-λ receptor subunits (IL-28RA and IL-10Rb) is required for IFN-λ signaling in CMT cells. CMT cells respond to IFN-λ via interferon-stimulated gene expression; however, our data suggest that this process might be independent of STAT activation. Our study demonstrated that IFN-λ enhances the migratory ability of P114 cells but decreases CMT-U27 cell migration. We show that this process is associated with the elevated gene expression of the extracellular matrix metalloproteinases and their inhibitors in a Akt and ERK-independent manner. However, we showed the minimal role of IFN-λ in CMT cell survival.

Our research has shed new light on the relationship between CMT and IFN-λ; nevertheless, further investigations on the role of INF-λ in CMT are needed.

## Figures and Tables

**Table 1:** Sequences of primers used for qPCR.

Gene Group	Gene	Sequence
Interferon-stimulated Genes	OAS-1	F: ATCTCCTGCCAGACACACAG
		R: GTGAAGCAGGTGGAGAACTC
	ISG-15	F: TCTGTGCCCCTGGAGGACTTGA
		R: TGCTGCTTCAGCTCTGATGCCA
	MX-1	F: GAATCCTGTACCCAATCATGTG
		R: TACCTTCTCCTCATATTGGCT
Matrix metalloproteinases	MMP-2	F: 5′ GGGACCACGGAAGACTATGA 3′
		R: 5′ ATAGTGGACATGGCGGTCTC 3′
	MMP-9	F: TGAGAACTAATCTCACTGACAAGCA
		R: GCTCGGCCACTTGAGTGTA
	MMP-13	F: CTCTTCTTCTCGGGAAACCA
		R: GCCTGGGGTAGTCTTTATCCA
Matrix metalloproteinases inhibitors	TIMP-1	F: CAGGGCCTGTACCTGTGC
		R: CCTGATGACGATTTGGGAGT
	TIMP-2	F: ATGAGATCAAGCAGATAAAGATGTTC
		R: GGAGGAAGGAGCCGTGTAG
	TIMP-3	F: TGCTGACAGGCCGCGT
		R: GCAGTTACAGCCCAGGTGA
Housekeeping gene	RPS19	F: GTTCTCATCGTAGGGAGCAAG
		R: CCTTCCTCAAAAAGTCTGGG

**Table 2:** Primary antibodies used for Western blot analysis.

Target Protein	Host	Dilution	Manufacturer Catalog Number
IL-28RA	rabbit	1:350	Abexxa abx322648
IL-10Rb	rabbit	1:150	Abexxa abx103170
phospho-STAT1	rabbit	1:1000	Biorbyt orb214616
phospho-STAT3	rabbit	1:1000	Biorbyt orb224010
phospho-Akt	rabbit	1:1000	Cell Signaling Technology #9271
Akt	rabbit	1:1000	Cell Signaling Technology #9272
phospho-ERK1/2	rabbit	1:1000	Cell Signaling Technology #4370
ERK1/2	rabbit	1:1000	Cell Signaling Technology #4695
β-actin	mouse	1:1000	Santa Cruz Biotechnology sc-47778
Lamin B	goat	1:1000	Santa Cruz Biotechnology sc-6216

## Data Availability

All data are available from the corresponding author.

## References

[1] Choi J.-W., Yoon H.-Y., Jeong S.-W. (2016). Clinical Outcomes of Surgically Managed Spontaneous Tumors in 114 Client-Owned Dogs. Immune Netw..

[2] Nunes F.C., Damasceno K.A., de Campos C.B., Bertagnolli A.C., Lavalle G.E., Cassali G.D. (2019). Mixed Tumors of the Canine Mammary Glands: Evaluation of Prognostic Factors, Treatment, and Overall Survival. Vet. Anim. Sci..

[3] Abdelmegeed S., Mohammed S. (2018). Canine Mammary Tumors as a Model for Human Disease (Review). Oncol. Lett..

[4] Vilhena H., Figueira A.C., Schmitt F., Canadas A., Chaves R., Gama A., Dias-Pereira P., Pastorinho M.R., Sousa A.C.A. (2020). Canine and Feline Spontaneous Mammary Tumours as Models of Human Breast Cancer. Pets as Sentinels, Forecasters and Promoters of Human Health.

[5] Soares M., Madeira S., Correia J., Peleteiro M., Cardoso F., Ferreira F. (2016). Molecular Based Subtyping of Feline Mammary Carcinomas and Clinicopathological Characterization. Breast.

[6] Ariyarathna H., Thomson N.A., Aberdein D., Perrott M.R., Munday J.S. (2020). Increased Programmed Death Ligand (PD-L1) and Cytotoxic T-Lymphocyte Antigen-4 (CTLA-4) Expression Is Associated with Metastasis and Poor Prognosis in Malignant Canine Mammary Gland Tumours. Vet. Immunol. Immunopathol..

[7] Urbano A.C., Nascimento C., Soares M., Correia J., Ferreira F. (2020). Clinical Relevance of the Serum CTLA-4 in Cats with Mammary Carcinoma. Sci. Rep..

[8] Nascimento C., Urbano A.C., Gameiro A., Ferreira J., Correia J., Ferreira F. (2020). Serum PD-1/PD-L1 Levels, Tumor Expression and PD-L1 Somatic Mutations in HER2-Positive and Triple Negative Normal-Like Feline Mammary Carcinoma Subtypes. Cancers.

[9] Amini P., Nassiri S., Ettlin J., Malbon A., Markkanen E. (2019). Next-Generation RNA Sequencing of FFPE Subsections Reveals Highly Conserved Stromal Reprogramming between Canine and Human Mammary Carcinoma. Dis. Model. Mech..

[10] Ettlin J., Clementi E., Amini P., Malbon A., Markkanen E. (2017). Analysis of Gene Expression Signatures in Cancer-Associated Stroma from Canine Mammary Tumours Reveals Molecular Homology to Human Breast Carcinomas. Int. J. Mol. Sci..

[11] Markkanen E. (2019). Know Thy Model: Charting Molecular Homology in Stromal Reprogramming Between Canine and Human Mammary Tumors. Front. Cell Dev. Biol..

[12] Amini P., Nassiri S., Malbon A., Markkanen E. (2020). Differential Stromal Reprogramming in Benign and Malignant Naturally Occurring Canine Mammary Tumours Identifies Disease-Modulating Stromal Components. Sci. Rep..

[13] Mucha J., Majchrzak K., Taciak B., Hellmén E., Król M. (2014). MDSCs Mediate Angiogenesis and Predispose Canine Mammary Tumor Cells for Metastasis via IL-28/IL-28RA (IFN-λ) Signaling. PLoS ONE.

[14] Stanifer M.L., Pervolaraki K., Boulant S. (2019). Differential Regulation of Type I and Type III Interferon Signaling. Int. J. Mol. Sci..

[15] Kotenko S.V., Gallagher G., Baurin V.V., Lewis-Antes A., Shen M., Shah N.K., Langer J.A., Sheikh F., Dickensheets H., Donnelly R.P. (2003). IFN-Λs Mediate Antiviral Protection through a Distinct Class II Cytokine Receptor Complex. Nat. Immunol..

[16] Sheppard P., Kindsvogel W., Xu W., Henderson K., Schlutsmeyer S., Whitmore T.E., Kuestner R., Garrigues U., Birks C., Roraback J. (2003). IL-28, IL-29 and Their Class II Cytokine Receptor IL-28R. Nat. Immunol..

[17] O’Brien T.R., Prokunina-Olsson L., Donnelly R.P. (2014). IFN-Λ4: The Paradoxical New Member of the Interferon Lambda Family. J. Interferon Cytokine Res..

[18] Lasfar A., Lewis-Antes A., Smirnov S.V., Anantha S., Abushahba W., Tian B., Reuhl K., Dickensheets H., Sheikh F., Donnelly R.P. (2006). Characterization of the Mouse IFN-λ Ligand-Receptor System: IFN-Λs Exhibit Antitumor Activity against B16 Melanoma. Cancer Res..

[19] Reuter A., Soubies S., Hartle S., Schusser B., Kaspers B., Staeheli P., Rubbenstroth D., Garcia-Sastre A. (2014). Antiviral Activity of Lambda Interferon in Chickens. J. Virol..

[20] Zhou P., Cowled C., Todd S., Crameri G., Virtue E.R., Marsh G.A., Klein R., Shi Z., Wang L.-F., Baker M.L. (2011). Type III IFNs in Pteropid Bats: Differential Expression Patterns Provide Evidence for Distinct Roles in Antiviral Immunity. J. Immunol..

[21] Quintana M.E., Cardoso N.P., Pereyra R., Barone L.J., Barrionuevo F.M., Mansilla F.C., Turco C.S., Capozzo A.V. (2020). Interferon Lambda Protects Cattle against Bovine Viral Diarrhea Virus Infection. Vet. Immunol. Immunopathol..

[22] Ichihashi T., Asano A., Usui T., Takeuchi T., Watanabe Y., Yamano Y. (2013). Antiviral and Antiproliferative Effects of Canine Interferon-Λ1. Vet. Immunol. Immunopathol..

[23] Kim D.-H., Park B.-J., Ahn H.-S., Go H.-J., Kim D.-Y., Kim J.-H., Lee J.-B., Park S.-Y., Song C.-S., Lee S.-W. (2021). Canine Interferon Lambda 3 Expressed Using an Adenoviral Vector Effectively Induces Antiviral Activity against Canine Influenza Virus. Virus Res..

[24] Fan W., Xu L., Ren L., Qu H., Li J., Liang J., Liu W., Yang L., Luo T. (2014). Functional Characterization of Canine Interferon-Lambda. J. Interferon Cytokine Res..

[25] Hubert M., Gobbini E., Couillault C., Manh T.-P.V., Doffin A.-C., Berthet J., Rodriguez C., Ollion V., Kielbassa J., Sajous C. (2020). IFN-III Is Selectively Produced by CDC1 and Predicts Good Clinical Outcome in Breast Cancer. Sci. Immunol..

[26] Finotti G., Tamassia N., Cassatella M.A. (2017). Interferon-Λs and Plasmacytoid Dendritic Cells: A Close Relationship. Front. Immunol..

[27] Israelow B., Narbus C.M., Sourisseau M., Evans M.J. (2014). HepG2 Cells Mount an Effective Antiviral Interferon-Lambda Based Innate Immune Response to Hepatitis C Virus Infection. Hepatology.

[28] Swider A., Siegel R., Eskdale J., Gallagher G. (2014). Regulation of Interferon Lambda-1 (IFNL1/IFN-Λ1/IL-29) Expression in Human Colon Epithelial Cells. Cytokine.

[29] Rodríguez Stewart R.M., Berry J.T.L., Berger A.K., Yoon S.B., Hirsch A.L., Guberman J.A., Patel N.B., Tharp G.K., Bosinger S.E., Mainou B.A. (2019). Enhanced Killing of Triple-Negative Breast Cancer Cells by Reassortant Reovirus and Topoisomerase Inhibitors. J. Virol..

[30] Lasfar A., Zloza A., Silk A.W., Lee L.Y., Cohen-Solal K.A. (2018). Interferon Lambda: Toward a Dual Role in Cancer. J. Interferon Cytokine Res..

[31] Lasfar A., Abushahba W., Balan M., Cohen-Solal K.A. (2011). Interferon Lambda: A New Sword in Cancer Immunotherapy. Clin. Dev. Immunol..

[32] Novak A.J., Grote D.M., Ziesmer S.C., Rajkumar V., Doyle S.E., Ansell S.M. (2008). A Role for IFN-Λ1 in Multiple Myeloma B Cell Growth. Leukemia.

[33] Lee S.-J., Lee E.-J., Kim S.-K., Jeong P., Cho Y.-H., Yun S.J., Kim S., Kim G.-Y., Choi Y.H., Cha E.-J. (2012). Identification of Pro-Inflammatory Cytokines Associated with Muscle Invasive Bladder Cancer; The Roles of IL-5, IL-20, and IL-28A. PLoS ONE.

[34] Lee S.-J., Lim J.-H., Choi Y.H., Kim W.-J., Moon S.-K. (2012). Interleukin-28A Triggers Wound Healing Migration of Bladder Cancer Cells via NF-ΚB-Mediated MMP-9 Expression Inducing the MAPK Pathway. Cell. Signal..

[35] Pingwara R., Witt-Jurkowska K., Ulewicz K., Mucha J., Tonecka K., Pilch Z., Taciak B., Zabielska-Koczywas K., Mori M., Berardozzi S. (2017). Interferon Lambda 2 Promotes Mammary Tumor Metastasis via Angiogenesis Extension and Stimulation of Cancer Cell Migration. J. Physiol. Pharmacol..

[36] Aresu L., Giantin M., Morello E., Vascellari M., Castagnaro M., Lopparelli R., Zancanella V., Granato A., Garbisa S., Aricò A. (2011). Matrix Metalloproteinases and Their Inhibitors in Canine Mammary Tumors. BMC Vet. Res..

[37] Klingemann H. (2018). Immunotherapy for Dogs: Running Behind Humans. Front. Immunol..

[38] Zmigrodzka M., Rzepecka A., Krzyzowska M., Witkowska-Pilaszewicz O., Cywinska A., Winnicka A. (2018). The Cyclooxygenase-2/Prostaglandin E2 Pathway and Its Role in the Pathogenesis of Human and Dog Hematological Malignancies. J. Physiol. Pharmacol..

[39] Bujak J.K., Pingwara R., Nelson M.H., Majchrzak K. (2018). Adoptive Cell Transfer: New Perspective Treatment in Veterinary Oncology. Acta Vet. Scand..

[40] Berraondo P., Sanmamed M.F., Ochoa M.C., Etxeberria I., Aznar M.A., Pérez-Gracia J.L., Rodríguez-Ruiz M.E., Ponz-Sarvise M., Castañón E., Melero I. (2019). Cytokines in Clinical Cancer Immunotherapy. Br. J. Cancer.

[41] Borden E.C. (2019). Interferons α and β in Cancer: Therapeutic Opportunities from New Insights. Nat. Rev. Drug Discov..

[42] Thul P.J., Åkesson L., Wiking M., Mahdessian D., Geladaki A., Ait Blal H., Alm T., Asplund A., Björk L., Breckels L.M. (2017). A Subcellular Map of the Human Proteome. Science.

[43] Uhlen M., Zhang C., Lee S., Sjöstedt E., Fagerberg L., Bidkhori G., Benfeitas R., Arif M., Liu Z., Edfors F. (2017). A Pathology Atlas of the Human Cancer Transcriptome. Science.

[44] Rajakylä K., Krishnan R., Tojkander S. (2017). Analysis of Contractility and Invasion Potential of Two Canine Mammary Tumor Cell Lines. Front. Vet. Sci..

[45] Maycotte P., Gearheart C.M., Barnard R., Aryal S., Mulcahy Levy J.M., Fosmire S.P., Hansen R.J., Morgan M.J., Porter C.C., Gustafson D.L. (2014). STAT3-Mediated Autophagy Dependence Identifies Subtypes of Breast Cancer Where Autophagy Inhibition Can Be Efficacious. Cancer Res..

[46] Avalle L., Pensa S., Regis G., Novelli F., Poli V. (2012). STAT1 and STAT3 in Tumorigenesis: A Matter of Balance. JAK-STAT.

[47] Furth P.A. (2014). STAT Signaling in Different Breast Cancer Sub-Types. Mol. Cell. Endocrinol..

[48] Król M., Pawłowski K.M., Dolka I., Musielak O., Majchrzak K., Mucha J., Motyl T. (2011). Density of Gr1-Positive Myeloid Precursor Cells, p-STAT3 Expression and Gene Expression Pattern in Canine Mammary Cancer Metastasis. Vet. Res. Commun..

[49] Lasfar A., Gogas H., Zloza A., Kaufman H.L., Kirkwood J.M. (2016). IFN-λ Cancer Immunotherapy: New Kid on the Block. Immunotherapy.

[50] Gao D., Yu X., Zhang B., Kong M., Fang Y., Cai Y., Zhu C., Zhao J., Li J. (2019). Role of Autophagy in Inhibiting the Proliferation of A549 Cells by Type III Interferon. Cell Biol. Int..

[51] Gao D., Zhao J., Li X., Xia Y., Cai Y., Pan J., Zhou H., Fang Y., Zhang S., Wen H. (2015). Interferon-Λ1 Suppresses Invasion and Enhances Autophagy in Human Osteosarcoma Cell. Int. J. Clin. Exp. Med..

[52] Mulcahy Levy J.M., Thorburn A. (2020). Autophagy in Cancer: Moving from Understanding Mechanism to Improving Therapy Responses in Patients. Cell Death Differ..

[53] García-Macia M., Santos-Ledo A., Caballero B., Rubio-González A., de Luxán-Delgado B., Potes Y., Rodríguez-González S.M., Boga J.A., Coto-Montes A. (2019). Selective Autophagy, Lipophagy and Mitophagy, in the Harderian Gland along the Oestrous Cycle: A Potential Retrieval Effect of Melatonin. Sci. Rep..

[54] Rabinowitz J.D., White E. (2010). Autophagy and Metabolism. Science.

[55] Wu D., Sanin D.E., Everts B., Chen Q., Qiu J., Buck M.D., Patterson A., Smith A.M., Chang C.-H., Liu Z. (2016). Type 1 Interferons Induce Changes in Core Metabolism That Are Critical for Immune Function. Immunity.

[56] Wang F., Zhang S., Jeon R., Vuckovic I., Jiang X., Lerman A., Folmes C.D., Dzeja P.D., Herrmann J. (2018). Interferon Gamma Induces Reversible Metabolic Reprogramming of M1 Macrophages to Sustain Cell Viability and Pro-Inflammatory Activity. EBioMedicine.

[57] Zhou Z., Hamming O.J., Ank N., Paludan S.R., Nielsen A.L., Hartmann R. (2007). Type III Interferon (IFN) Induces a Type I IFN-Like Response in a Restricted Subset of Cells through Signaling Pathways Involving Both the Jak-STAT Pathway and the Mitogen-Activated Protein Kinases. J. Virol..

[58] Guenterberg K.D., Grignol V.P., Raig E.T., Zimmerer J.M., Chan A.N., Blaskovits F.M., Young G.S., Nuovo G.J., Mundy B.L., Lesinski G.B. (2010). Interleukin-29 Binds to Melanoma Cells Inducing Jak-STAT Signal Transduction and Apoptosis. Mol. Cancer Ther..

[59] Dien J., Amin H.M., Chiu N., Wong W., Frantz C., Chiu B., Mackey J.R., Lai R. (2006). Signal Transducers and Activators of Transcription-3 Up-Regulates Tissue Inhibitor of Metalloproteinase-1 Expression and Decreases Invasiveness of Breast Cancer. Am. J. Pathol..

[60] Zhang F., Wang Z., Fan Y., Xu Q., Ji W., Tian R., Niu R. (2015). Elevated STAT3 Signaling-Mediated Upregulation of MMP-2/9 Confers Enhanced Invasion Ability in Multidrug-Resistant Breast Cancer Cells. Int. J. Mol. Sci..

